# Genetic Variation in Iron Metabolism Is Associated with Neuropathic Pain and Pain Severity in HIV-Infected Patients on Antiretroviral Therapy

**DOI:** 10.1371/journal.pone.0103123

**Published:** 2014-08-21

**Authors:** Asha R. Kallianpur, Peilin Jia, Ronald J. Ellis, Zhongming Zhao, Cinnamon Bloss, Wanqing Wen, Christina M. Marra, Todd Hulgan, David M. Simpson, Susan Morgello, Justin C. McArthur, David B. Clifford, Ann C. Collier, Benjamin B. Gelman, J. Allen McCutchan, Donald Franklin, David C. Samuels, Debralee Rosario, Emily Holzinger, Deborah G. Murdock, Scott Letendre, Igor Grant, for the CHARTER Study Group

**Affiliations:** 1 Department of Genomic Medicine, Lerner Research Institute, Cleveland Clinic Foundation, Cleveland, Ohio, United States of America; 2 Department of Molecular Medicine, Cleveland Clinic Lerner College of Medicine of Case Western Reserve University, Cleveland, Ohio, United States of America; 3 Department of Biomedical Informatics, Vanderbilt University School of Medicine, Nashville, Tennessee, United States of America; 4 Department of Neurology, University of California San Diego, San Diego, California, United States of America; 5 Scripps Genomic Medicine, Scripps Translational Science Institute, and Scripps Health, La Jolla, California, United States of America; 6 Department of Medicine, Vanderbilt University School of Medicine, Nashville, Tennessee, United States of America; 7 Department of Neurology, University of Washington, Seattle, Washington, United States of America; 8 Department of Neurology, Icahn School of Medicine of Mt. Sinai, New York, New York, United States of America; 9 Department of Neurology, Johns Hopkins University, Baltimore, Maryland, United States of America; 10 Department of Neurology, Washington University, St. Louis, Missouri, United States of America; 11 Department of Medicine, University of Washington, Seattle, Washington, United States of America; 12 Department of Pathology, University of Texas Medical Branch, Galveston, Texas, United States of America; 13 Department of Medicine, University of California San Diego, San Diego, California, United States of America; 14 HIV Neurobehavioral Research Center & CHARTER Center, University of California San Diego, San Diego, California, United States of America; 15 Department of Molecular Physiology and Biophysics and Center for Human Genetics Research, Vanderbilt University School of Medicine, Nashville, Tennessee, United States of America; 16 Department of Psychiatry, University of California San Diego, San Diego, California, United States of America; Lady Davis Institute for Medical Research/McGill University, Canada

## Abstract

HIV sensory neuropathy and distal neuropathic pain (DNP) are common, disabling complications associated with combination antiretroviral therapy (cART). We previously associated iron-regulatory genetic polymorphisms with a reduced risk of HIV sensory neuropathy during more neurotoxic types of cART. We here evaluated the impact of polymorphisms in 19 iron-regulatory genes on DNP in 560 HIV-infected subjects from a prospective, observational study, who underwent neurological examinations to ascertain peripheral neuropathy and structured interviews to ascertain DNP. Genotype-DNP associations were explored by logistic regression and permutation-based analytical methods. Among 559 evaluable subjects, 331 (59%) developed HIV-SN, and 168 (30%) reported DNP. Fifteen polymorphisms in 8 genes (*p*<0.05) and 5 variants in 4 genes (*p*<0.01) were nominally associated with DNP: polymorphisms in *TF*, *TFRC*, *BMP6*, *ACO1*, *SLC11A2*, and *FXN* conferred reduced risk (adjusted odds ratios [ORs] ranging from 0.2 to 0.7, all *p*<0.05); other variants in *TF*, *CP*, *ACO1*, *BMP6*, and *B2M* conferred increased risk (ORs ranging from 1.3 to 3.1, all *p*<0.05). Risks associated with some variants were statistically significant either in black or white subgroups but were consistent in direction. *ACO1* rs2026739 remained significantly associated with DNP in whites (permutation *p*<0.0001) after correction for multiple tests. Several of the same iron-regulatory-gene polymorphisms, including *ACO1* rs2026739, were also associated with severity of DNP (all *p*<0.05). Common polymorphisms in iron-management genes are associated with DNP and with DNP severity in HIV-infected persons receiving cART. Consistent risk estimates across population subgroups and persistence of the *ACO1* rs2026739 association after adjustment for multiple testing suggest that genetic variation in iron-regulation and transport modulates susceptibility to DNP.

## Introduction

The success of combination antiretroviral therapy (cART) in human immunodeficiency virus (HIV) infection has focused attention on addressing long-term complications that reduce quality of life, such as peripheral neuropathy [1]. Peripheral neuropathy in HIV infection is a distal symmetric, often painful, sensory polyneuropathy associated with the virus itself and/or to toxic effects of certain antiretroviral drugs, particularly the dideoxy-nucleoside reverse-transcriptase inhibitors (so-called “D-drug” NRTIs) such as didanosine and stavudine [2], [3]. Clinically and histopathologically, neuropathy due to HIV infection in the pre-cART era and the neuropathy associated with cART toxicity are usually indistinguishable; both are encompassed by the term “HIV sensory neuropathy” (HIV-SN). HIV-SN may first manifest or worsen upon initiation of cART and is associated with sensory loss, paresthesias, and distal neuropathic pain (DNP) or dysesthesias. The known mitochondrial toxicity of D-drugs and dysregulated inflammation have both been implicated in its pathogenesis, but the pathophysiology of HIV-SN remains incompletely understood [2], [4]–[8].

Development of DNP is particularly disabling [9]–[11], because the symptoms respond poorly to analgesic medications that are used to treat other types of neuropathic pain [1], [12]–[15]. HIV-SN therefore has an adverse impact on quality of life, social and emotional functioning, and neuropsychological test performance, and DNP in particular contributes to depression, physical deconditioning, and non-adherence to treatment [10], [11], [16]–[18]. Although substitution of less toxic antiretroviral drugs lowers the risk of HIV-SN, older D-drugs like stavudine are likely to remain in use in low-resource settings for some time as part of generic, fixed-dose cART regimens, and the study of HIV-SN and DNP remains very relevant [19], [20]. Improved understanding of the biological mechanisms underlying painful HIV-SN may lead to better ways of categorizing and therapeutically targeting DNP [21]–[23].

Iron is a critical micronutrient for metabolically active cells such as neurons, and a carefully controlled supply of iron is essential for mitochondrial function, axonal transport, and myelination [24]–[26]. Iron transport also plays an important role in regulating macrophage-mediated inflammation via the hepcidin pathway [27]–[30]. Hepcidin, a peptide hormone which is synthesized in the liver in response to inflammatory stimuli, leads to iron sequestration within the reticuloendothelial cell (macrophage-monocyte) compartment, potentiating macrophage oxidative killing via Fenton chemistry while reducing availability of iron to micro-organisms. We previously reported associations between iron-loading, single-nucleotide polymorphisms (SNPs) in the hemochromatosis (*HFE*) gene and reduced risk of HIV-SN in HIV-infected individuals exposed to D-drug-containing cART [31]. While improved cellular and mitochondrial iron delivery might play a protective role in individuals with *HFE* variants, in whom the action of hepcidin is reduced, these associations could also be explained by the known linkage of *HFE* to the HLA Class I locus and thereby to other immunomodulatory haplotypes [31], [32]. Studies of non-HLA-linked genetic variants that are prevalent in racially diverse populations, unlike *HFE* gene variants, are needed to determine whether iron-transport is of fundamental importance in susceptibility to HIV-SN and neuropathic symptoms like DNP.

We hypothesized that like *HFE* variants, common variants in other iron-management but non-HLA-Class I-linked genes modulate susceptibility to cART-associated HIV-SN and DNP in HIV-infected individuals, and that these genes impact neuropathy phenotypes independent of race/ethnicity and disease-related factors. This study assessed the role of variants in key iron-transport and iron-regulatory genes (henceforth termed “the ferrome” for brevity) in susceptibility to neuropathy phenotypes in a racially diverse, HIV-infected population, the CNS HIV Antiretroviral Therapy Effects Research (CHARTER) Cohort. CHARTER is the largest U.S.-based prospective study of neurological complications in HIV/AIDS in the cART era [33]. Particular strengths of CHARTER include its clear definition of neurological phenotypes and sensitive and specific methods of ascertaining HIV-SN. This analysis specifically targeted genes that encode components of the nuclear and mitochondrial ferrome, for which SNP genotypes were available from a genomic study conducted previously in CHARTER. In addition to several SNPs that were nominally associated with DNP, a common variant (rs2026739) in the cytoplasmic aconitase gene (*ACO1*) was identified to be statistically associated with DNP after multiple-testing correction.

## Patients and Methods

### Study population

The CHARTER Study is a prospective, observational study conducted at six outpatient centers within the U.S.: Johns Hopkins University, Baltimore, Maryland; Mount Sinai School of Medicine, New York, NY; the University of California, San Diego, CA; the University of Texas Medical Branch, Galveston, TX; the University of Washington, Seattle, WA; and Washington University, St Louis, MO. Data collection was standardized across centers and employed a protocol of comprehensive neuromedical, neurobehavioral, and laboratory assessments, including: nadir CD4+ T-cell count, cART history, D-drug exposure, history of a major depressive disorder, and demographic information (from structured interviews), as well as current CD4+ T-cell count, HIV plasma viral load, and hepatitis C viral serology (from laboratory data). Further details regarding CHARTER Study eligibility and enrollment procedures have been reported previously [1]. A subset of CHARTER Study subjects (N = 579) was recruited between 2003 and 2007 to a cross-sectional genomic sub-study of peripheral neuropathy. Genetic study eligibility criteria included the ability to provide details of cART use and to undergo a structured interview and examination for signs/symptoms of HIV-SN; individuals with active opportunistic infections, uncontrolled psychiatric disorders, or who were unable to cooperate with detailed evaluations over the course of a full day were excluded.

#### Ethics Statement

The CHARTER Study and this study were approved by the University of California-San Diego Human Research Protections Program (Biomedical Institutional Review Board) and by the Institutional Review Boards (IRBs) of the following participating CHARTER institutions: the Johns Hopkins University School of Medicine IRB, Icahn School of Medicine at Mount Sinai IRB, University of Texas (Medical Branch) IRB, University of Washington School of Medicine IRB, and Washington University -St. Louis School of Medicine IRB. The present study was conducted according to the principles expressed in the Declaration of Helsinki. All study subjects whose data are reported herein provided written informed consent.

### Ascertainment of neuropathy and distal neuropathic pain

Standardized, targeted neurological examinations were performed by physicians and nurses trained in neurological AIDS disorders. HIV-SN and DNP were identified using criteria published previously [1], [10]. Briefly, participants were queried at baseline and each follow-up visit about symptoms of HIV-SN and DNP, specifically, tingling, or burning, aching, shooting, or stabbing pain in a bilateral, predominantly distal distribution (*e.g*., in the fingers and toes only, extending to the ankles or wrists, or extending to the knees or elbows). If subjects endorsed tingling in this distribution, they were considered to have paresthesias; if they endorsed any of the pain symptoms in this distribution, they were considered to have a positive self-report of DNP; loss of sensation in the lower extremities was also assessed on examination. HIV-SN cases were defined by the bilateral presence of at least one of the following signs at any visit: diminished vibration sense, reduced sharp-dull discrimination in the feet and toes, reduced ankle reflexes, and DNP cases by the above-mentioned pain symptoms; controls did not have any of these signs or symptoms of peripheral nerve disease. DNP was also classified into the following 5 severity levels by structured survey; none, slight (occasional, fleeting), mild (frequent), moderate (frequent, disabling) or severe (constant, daily, disabling, and requiring treatment with analgesics or other medications).

### Genomic DNA isolation and gene selection

Genomic DNA was isolated from whole blood samples using PUREGENE (Gentra Systems Inc., Minneapolis, MN, USA). Samples were subjected to whole-genome nuclear genotyping using the Affymetrix Genome-Wide Human SNP Array 6.0 platform (Affymetrix, Inc., Santa Clara, CA). Among 579 extracted samples, 560 yielded analyzable genotypes that passed quality-control filters using the Platform for the Analysis, Translation, and Organization of large scale data (PLATO) [34]. Sample genotyping efficiency was 95%. Variants with less than 95% genotyping efficiency or minor allele frequencies (MAFs) less than 1% in the study population were excluded from analysis.

Genes reported in the published literature to be associated with iron metabolism and/or neurological phenotypes were searched in the publicly available Online Mendelian Inheritance in Man database (http://www.ncbi.nlm.nih.gov/omim). We then identified 20 genes based on their well-recognized direct or indirect involvement in iron metabolism, transport, storage, or regulation (*e.g*., the hepcidin pathway). SNPs mapping to the following iron-regulatory and transport genes were selected for this exploratory analysis based on their inclusion in the Affymetrix Genome-Wide Human SNP Array 6.0: *HFE*, *HFE2*, *SLC40A1*, *SLC11A1*, *HAMP*, *TF*, *TFRC*, *TFR2*, *BMP2*, *BMP6*, *CP*, *SLC11A2*, *FXN*, *FTMT*, *FTH1*, *ACO1*, *ACO2*, *B2M* and *ATP13A2*. Nineteen of these genes were covered by the Affymetrix 6.0 chip, and the platform provided evaluable genotypic information for 192 candidate SNPs (Table S1). Many of these iron-related genes/SNPs have also been previously reported to play a role in iron transport within the nervous system, and genes previously linked to neurodegenerative diseases and neural differentiation were included [35]. Based on the common disease-common variant hypothesis, this list was further narrowed to those SNPs with MAFs of at least 5% in one or both of the largest subsets of the CHARTER study population (non-Hispanic blacks or non-Hispanic whites, henceforth referred to as blacks or whites, respectively) [36]. Using a typical analysis strategy that has been applied in numerous previous studies, we used all available genotyped SNPs in those genes, though some of them may be correlated due to LD structure.

### Statistical methods

#### General approach

The role of iron-related genes in susceptibility to cART-associated HIV-SN and DNP was evaluated by both multivariable logistic regression and permutation analysis. Since the SNPs analyzed could not be assumed to be independent, Bonferroni adjustment of statistical tests would almost certainly overcorrect and result in unacceptable Type II error; permutation-based analysis is a complementary statistical method that accounts for multiple statistical tests, gene size, and linkage disequilibrium [37], [38]. Specifically, the observed *p*-value from the real dataset is compared to the distribution of *p*-values generated from 1000 permutated sample sets which randomly switch case/control labels while maintaining the same case/control ratio [37]. An empirical *p*-value is generated by the number of permutation sets whose *p*-values are less than the observed *p*-value divided by 1000.

Population stratification was also evaluated in our analyses by adjustment for four principal components variables (PCs) representing ancestry-related genetic information, which were generated from genome-wide genotype data in CHARTER and used as covariates in multivariate logistic regression models, in addition to adjustment for self-reported race/ethnicity. Logistic regression models stratified by self-reported race/ethnicity were also explored (black or white; other categories were omitted due to insufficient numbers). The degree of potential overlap between individual PCs was also evaluated by plotting these variables against one another (Figure S1).

The following neuropathy outcomes were analyzed: presence of *at least 1 sign* of HIV-SN, *at least 2 signs* of HIV-SN, and DNP. Each outcome was analyzed as a dichotomous variable. Potential confounders were identified by univariable logistic regression to calculate unadjusted odds ratios (ORs) and by prior published associations of these variables with HIV-SN, neuropathy symptoms (loss of sensation, paresthesias, and/or dysesthesias/DNP), or DNP alone in CHARTER.[1], [9], [33]. Significant variables were then included in all multivariable logistic models with or without race-stratification. These phenotype-specific covariates were as follows: age (years), cumulative D-drug exposure (months), CD4+ T-cell nadir, log_10_[plasma HIV RNA], hepatitis C virus (HCV) serologic status (positive/negative), protease inhibitor use (0 or ≥1 protease inhibitor), current or prior major depressive disorder (yes/no), self-reported race (in non-stratified models), and the 4 genomic sequence-derived ancestry PC variables in HIV-SN analyses; and age, cumulative D-drug exposure, CD4+ T-cell nadir, log_10_[plasma HIV RNA], self-reported race (in non-stratified analyses), and the 4 PC variables in analyses of neuropathy symptoms and DNP. In order to account for the effects of diabetes in some subjects who might also be on treatment for this condition, we used a more stringent blood glucose criterion for ascertaining diabetes than is normally used for non-fasting blood glucose levels (≥126 mg/dL, rather than ≥200 mg/dL). By this criterion, 66 subjects (11.8%) were coded as diabetic. Since additional adjustment for diabetes by this method did not alter *p*-values appreciably for any of the studied SNPs, we did not include this covariate in final regression models. (Hemoglobin A1c levels, which would have allowed for more accurate diabetes diagnosis, were available in <10% subjects, and blood glucose values were missing in 6 subjects.)

The reported associations of genetic variants with presence or absence of HIV-SN or DNP made no genetic model assumptions regarding dominant or recessive allelic effects (genotypes were coded as 0, 1, or 2, based on the number of minor alleles present); all SNPs in the genes studied were bi-allelic.

For analysis of DNP severity, genotypes were dichotomized (variant alleles present or absent) to optimize power, and *p-*values were obtained using Pearson's chi-square test or, for those SNPs with fewer than 5 observations per cell, Fisher's exact test.

#### Multivariable single-SNP analyses

Statistical tests were performed using publicly available R software (http://www.r-project.org/). For each phenotype, logistic regression was used for single-marker association tests, while adjusting for phenotype-specific covariates in addition to race as a categorical covariate and ancestry PCs, as discussed above [1], [9]. Additional adjustment for cART-naïve status beyond inclusion of D-drug exposure and HIV viral load did not significantly alter results; hence, this variable was not included in multivariable models. PC variables were computed in advance based on genome-wide genotype data available in all 560 CHARTER study participants. Permutation tests were conducted by randomizing case/control labels in multivariable models while keeping the same numbers of cases and controls as in the original dataset. We generated 1000 permutation datasets. An empirical *p*-value was computed for each SNP in each phenotype, according to *p*_emp_ = #{*P*(π) <*P*(real)/1000, where *P*(π) is the *p*-value in the π^th^ permutation [38], [39].

**Figure 1:**
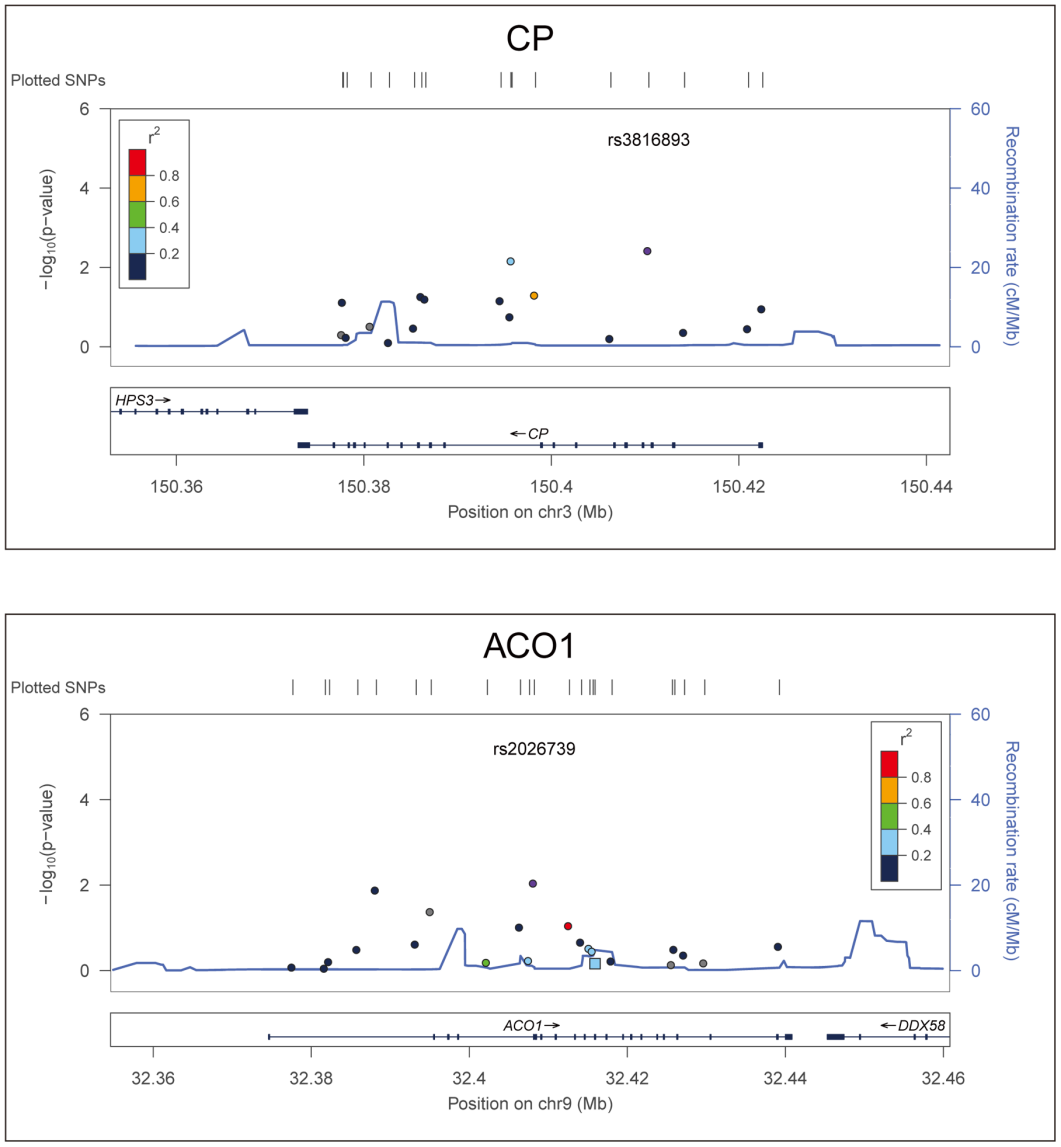
Representative Linkage Disequilibrium (LD) plot. The LD plot for two representative SNPs that were statistically significantly associated with neuropathic pain in the CHARTER cohort is displayed: *CP* rs3816893 and *ACO1* rs2026739. Each plot shows the degree of LD between the SNP of interest (rs number) and all other SNPs analyzed in the same gene, color-coded according to the r^2^ value (correlation of frequencies in this sample). The LD is based on HapMap Phase II CEU data. Association *p*-values are displayed on the y-axis and chromosomal distance (Mb) on the x-axis.

Results of association tests for two key genes, *CP* and *ACO1*, with neuropathic pain were depicted using the LocusZoom tool (Figure 1, generated using HapMap Phase II CEU) [40].

The *p*-values for all statistical tests were two-tailed, and for this exploratory analysis, the value of α (statistical significance) was set at 0.05 to identify SNPs of potential interest in this exploratory study. A Bonferroni correction for multiple statistical tests was also applied by multiplying *p*-values obtained for each association test by 192, the number of SNPs evaluated to identify more robust associations.

## Results

Genotypes from the CHARTER genome study were available for 192 SNPs encompassing 19 iron-regulatory or iron-transport-related (ferrome) genes queried (Table S1): *HFE*, *HJV*, *FPN1*, *SLC11A1*, *HAMP*, *TF*, *TFRC*, *TFR2*, *BMP2*, *BMP6*, *CP*, *SLC11A2*, *FXN*, *FTMT*, *FTH1*, *ACO1*, *ACO2*, *B2M*, and *ATP13A2*. Genotypes at the *HEPH* locus on the X chromosome, which encodes a ceruloplasmin-like, membrane-bound ferroxidase that is important in transmembrane iron transport, and genotypes at the *HFE* C187G locus (previously studied by the authors in HIV-infected subjects) were not covered by the Affymetrix Human SNP Array 6.0 platform. The CHARTER genomic study provided genotypes at all other loci of interest in >99% of subjects; of 559 evaluable individuals, 21% were women, and the mean age of the population was 44 years (range 21–68). Self-reported race/ethnicity was 43% (n = 242) black, 44% (n = 247) white, 11% (n = 58) Hispanic, and 2% Asian or “Other” (n = 11). Due to insufficient power for analyses in subjects of Hispanic, Asian or “Other” ancestry, only analyses performed in non-Hispanic blacks, whites, or the entire CHARTER genetic study population are reported. As shown in Figure S1, the 4 genome-derived PC variables in multivariable models of neuropathic pain accurately represented distinct ancestral strata in this study sample.

Demographic and HIV disease characteristics of subjects with and without HIV-SN are presented in Table 1. Among 559 evaluable subjects, 331 (59%) had at least one sign of HIV-SN, and 160 (29%) exhibited more severe HIV-SN (at least two signs). Fifty percent of the study population (281 subjects) reported at least one neuropathic symptom, including paresthesias, loss of sensation, dysesthesias, and/or DNP in both lower extremities. Ascertainment of dysesthesias correlated so closely with DNP that only results for DNP will be discussed henceforth. A total of 168 subjects (30% of this study sample) experienced DNP of some degree of severity. CHARTER study subjects with HIV-SN (at least one sign) and those who reported neuropathic symptoms and/or DNP were older than subjects without these complications (*p*<0.01). No significant differences were noted with regard to educational level (*data not shown*), self-reported race/ethnicity, or sex, but subjects reporting DNP tended to be female (26% *vs.* 19%, *p* = 0.06). Individuals with HIV-SN of any severity and those who reported neuropathy symptoms other than DNP had a lower CD4 nadir than corresponding controls [median (IQR) for cases *vs.* controls, respectively, were 112 (31, 246) *vs.* 242 (125, 391) cells/µL, *p*<0.01 for at least one HIV-SN sign; 107 (23, 214) *vs.* 200 (70, 350), *p*<0.01 for at least two signs; and 151 (37, 275) *vs.* 198 (60, 348), *p*<0.01 for neuropathy symptoms]. Median viral load was lower among HIV-SN cases than controls [median (IQR) 1.7 (1.7, 3.3) *vs.* 2.6 (1.7, 4.2) log_10_ (HIV RNA copies/mL), respectively, *p*<0.01] but not statistically different between subjects with or without neuropathy symptoms and DNP. Substantially fewer individuals with HIV-SN and DNP were cART-naïve as compared to controls (6% of cases with at least one sign of HIV-SN *vs.* 29% of controls; 8% of DNP cases *vs*. 19% of controls, *p*<0.01 for both). Cumulative D-drug exposure was also higher among cases than controls in all outcome categories, including DNP [*e.g.*, median (IQR) 14 (0, 53) *vs.* 0 (0, 21) months for DNP]; all *p*-values<0.01]. HIV-SN of any severity was associated with the use of protease inhibitors (53% current use in subjects with at least one sign *vs.* 31% of controls, *p*<0.01. Co-infection with hepatitis C virus (HCV) was more common among HIV-SN cases (28% of cases with at least one sign *vs*. 19% of controls; 31% of cases with at least two signs *vs.* 22% of controls, *p*<0.05 for both). History of a major depressive disorder was more frequently reported by subjects without HIV-SN (46%) than among HIV-SN cases (36%, *p*<0.05) and was slightly more common among individuals with DNP.

**Table 1:** Characteristics of neuropathy cases and controls in the CHARTER genetic study population.

Baseline Characteristic	HIV-SN, ≥1 sign		*p*-value*	HIV-SN, ≥2 signs		*p-*value*	Neuropathy Symptoms†		*p*- value*	Neuropathic Pain		*p*-value*
	Cases N = 331	Controls N = 228		Cases N = 160	Controls N = 399		Cases N = 281	Controls N = 274		Cases N = 168	Controls N = 390	
Age, mean±SD	46±8	40±8	<0.01	42±8	47±8	<0.01	45±8	42±8	<0.01	46±8	43±9	<0.01
Race/ethnicity, %			0.10			0.80			0.23			0..30
*Black*	46	40		43	43		40	47		38	46	
*White*	44	44		46	44		48	40		49	42	
*Hispanic*	9	13		10	11		10	11		11	10	
*Other*	1	3		1	2		2	2		2	2	
Sex, % women	19	23	0.23	18	22	0.23	23	18	0.13	26	19	0.06
CD4+ T-cell nadir,cells/uL, median(IQR)	112 (31, 246)	242 (125, 391)	<0.01	107 (23, 214)	200 (70, 350)	<0.01	151 (37, 275)	198 (60, 348)	<0.01	139 (56, 267)	187 (54, 325)	0.09
Log_10_ [HIV RNA copies/mL], median (IQR)	1.7 (1.7, 3.3)	2.6 (1.7, 4.2)	<0.01	1.7 (1.7, 3.0)	2.3 (1.7, 4.1)	<0.01	1.7 (1.7, 3.4)	2.2 (1.7, 4.0)	0.09	1.7 (1.7, 3.6)	2.0 (1.7, 3.9)	0.31
cART-naïve, %	6	29	<0.01	3	20	<0.01	11	20	<0.01	8	19	<0.01
Current PI use, % yes	53	31	<0.01	56	40	<0.01	48	40	0.14	46	44	0.49
Cumulative D-drug use, median mos.,(IQR)	14 (0, 53)	0 (0, 21)	<0.01	17 (0, 48)	0 (0, 36)	<0.01	11 (0, 41)	0 (0, 43)	0.01	12 (0, 48)	0 (0, 36)	<0.01
HCV positive, %	28	19	<0.05	31	22	<0.05	24	25	0.73	24	25	0.78
Current/prior major depression, %	36	46	<0.05	32	43	<0.05	42	39	0.43	43	39	0.39

*Abbreviations: SD*, standard deviation; *HIV-SN*, HIV sensory neuropathy; *cART*, combination antiretroviral therapy; *PI*, protease inhibitor; *HCV*, hepatitis C virus; *mos*., months; *IQR*, interquartile range.

† Neuropathy symptoms included bilateral paresthesias, dysesthesias/neuropathic pain, and/or loss of sensation in the lower extremities.

*p-values<0.05 are statistically significant.

Results of multivariable logistic regression and permutation analyses evaluating associations of selected SNPs with HIV-SN phenotypes, including DNP, are summarized in Table 2, adjusted for potential confounders, including self-reported race and 4 PC variables that captured racial admixture. Twelve SNPs in 8 iron-related genes were associated with at least one sign of HIV-SN (*p<*0.05) by permutation analyses, as compared with 13 SNPs in the same 8 genes analyzed by logistic regression. One variant (rs1049296 in the gene *TF*) was also associated with HIV-SN with a *p*-value<0.01. One variant in the *HFE* gene was also associated with reduced risk of HIV-SN in this population, replicating a prior association of this gene with reduced risk of HIV-SN [31]. More severe HIV-SN was associated with 10 SNPs in 7 iron-related genes (*p*<0.05) and with 4 SNPs in 4 genes (*p*<0.01) by permutation analysis. Fourteen SNPs in 8 iron-regulatory genes were significantly associated with DNP (*p*<0.05) by logistic regression and 15 SNPs by permutation analysis, including two variants (rs3816893 in the gene *CP* and rs2026739 in the gene *ACO1*). These SNPs were also associated with more severe HIV-SN (at least two signs, *data not shown*). Eighteen SNPs in 6 genes were associated in permutation analysis with the presence of one or a combination of uncomfortable neuropathy symptoms (paresthesias, loss of sensation, or dysesthesias/DNP, *p*<0.05). Four variants in 2 of these genes, including *CP* rs3816893 and *CP* rs13072552, were strongly associated with neuropathy symptoms by logistic regression (all *p*-values<0.01) as well as by permutation analysis (*p*<0.0001 for both SNPs mentioned), thereby also meeting criteria for significance after correction for multiple testing.

**Table 2:** Summary of significant results from multivariable analyses of peripheral neuropathy in CHARTER subjects.

Neuropathy Phenotype	*p*-values from Logistic regression*		Empirical *p*-values from Permutation*	
	No. of Significant genes (SNPs)			
	*p*<0.05	*p*<0.01	*p_emp_*<0.05	*p_emp_*<0.01
HIV-SN, ≥1 sign	8 (13)	1 (1)	8 (12)	(0)
HIV-SN, ≥2 signs	7 (12)	4 (4)	7 (10)	4 (4)
Neuropathic pain	8 (14)	4 (5)	8 (15)	4 (5)
≥1 Neuropathy symptom(s)¥	7 (18)	2 (4)	6 (18)	2 (4)

*Models shown were adjusted for the following covariates: age, cumulative D-drug exposure, CD4^+^ T-cell nadir, plasma HIV RNA concentration, self-reported race, protease inhibitor use, HCV status, history of major depression, and 4 ancestry by principal components (for HIV-SN); first five of the same factors plus principal components (for neuropathic pain or neuropathy symptoms).

¥ Includes presence of paresthesias, loss of sensation, and/or dysesthesias/neuropathic pain.

Analyses were performed with no genetic model assumptions (genotypes at each locus were coded as 0, 1, or 2 minor alleles).

*Abbreviations:* HIV-SN, HIV-sensory neuropathy.

A summary of iron-regulatory gene variants significantly associated in multivariable-adjusted analyses with DNP in CHARTER subjects stratified by self-reported race, with estimated ORs and 95% confidence intervals (CIs), is presented in Table 3. Eight iron-regulatory or iron-transport-related genes, *TF*, *CP*, *TFRC*, *BMP6*, *ACO1*, *FXN*, *SLC11A2*, and *B2M* were associated with DNP. Five SNPs in 4 iron regulatory genes, *TF* rs2718796, *CP* rs13072552 and rs3816893, *ACO1* rs2026739, and *B2M* rs16966334, were associated with increased risk in the combined CHARTER population by both logistic regression and permutation analysis in the entire population, with ORs ranging from 1.5 to 3.1 (all *p*<0.01). Race-stratified analyses revealed, however, that statistically significant associations for some of these SNPs occurred primarily within black or white subgroups. Point estimates for the associations of *CP* rs3816893 and *B2M* rs16966334 with DNP were greater in blacks than in whites and statistically significant only in blacks [OR 2.6 (95% CI 1.3–5.0) and 2.8 (95% CI 1.3–6.0), respectively, both *p*<0.01], while those for *ACO1* rs2026739 and *B2M* rs1901531 were statistically significant among whites only [OR 2.3 (95% CI 1.4–3.7, *p*<0.0001) and OR 2.2 (95% CI 1.3–3.8, *p*<0.01), respectively]. With the exception of *B2M* rs1901531, point estimates for ORs associating SNPs with DNP in this population were consistent in direction and very similar in magnitude across race-stratified analyses (black and white subgroups). Several additional SNPs were nominally associated with DNP by logistic regression as well as permutation-based methods of analysis, including *FXN* rs3793451 (all *p*<0.05). Two variants, *TF* rs8177306 and *ACO1* rs4495514, could not be analyzed in whites due to insufficient numbers of variant alleles.

**Table 3:** Genetic variants associated with neuropathic pain.

SNP	Gene	All Subjects (390 Cases/168 Controls)			White (163 Cases/83 Controls)			Black (179 Cases/63 Controls)		
		OR (95% CI)	*p*	*p*_emp_	OR (95% CI)	*p*	*p*_emp_	OR (95% CI)	*p*	*p*_emp_
rs2718796_g	*TF*	3.1 (1.4–7.3)	0.007	0.003	5.0 (1.1–28.1)	0.045	0.026	3.0 (0.7–12.3)	0.118	0.055
rs8177306_g	*TF*	0.4 (0.2–0.9)	0.023	0.017	–	–	–	0.5 (0.2–1.0)	0.060	0.059
rs13072552_t	*CP*	1.6 (1.1–2.4)	0.007	0.008	1.5 (0.7–3.1)	0.292	0.280	1.5 (1.0–2.4)	0.054	0.071
rs13075921_c	*CP*	1.6 (1.0–2.4)	0.048	0.035	1.4 (0.7–2.8)	0.343	0.358	2.1 (1.0–4.0)	0.037	0.049
rs3816893_t	*CP*	1.9 (1.2–3.0)	0.004	0.002	1.6 (0.8–3.3)	0.165	0.151	2.6 (1.3–5.0)	0.005	0.006
rs480760_t	*TFRC*	0.6 (0.4–0.9)	0.042	0.042	0.8 (0.2–2.1)	0.660	0.678	0.7 (0.0–1.1)	0.098	0.094
rs270388_t	*BMP6*	1.3 (1.0–1.8)	0.057	0.049	1.2 (0.7–2.2)	0.430	0.448	1.2 (0.2–1.9)	0.323	0.314
rs267202_a	*BMP6*	0.8 (0.6–1.0)	0.050	0.066	0.8 (0.5–1.3)	0.414	0.424	0.7 (0.5–1.1)	0.162	0.182
rs267206_c	*BMP6*	1.4 (1.0–2.0)	0.029	0.034	1.5 (0.9–2.6)	0.098	0.118	1.4 (0.9–2.2)	0.173	0.196
rs7033149_g	*ACO1*	1.6 (1.1–2.4)	0.013	0.012	1.7 (0.9–3.2)	0.118	0.128	1.7 (1.0–2.9)	0.044	0.046
rs4495514_t	*ACO1*	0.4 (0.2–0.9)	0.040	0.036	–	–	–	0.5 (0.2–1.1)	0.118	0.112
rs2026739_g	*ACO1*	1.5 (1.1–2.0)	0.009	0.007	2.3 (1.4–3.7)	0.001	<0.0001	1.3 (0.8–2.1)	0.233	0.228
rs3793451_t	*FXN*	0.4 (0.2–0.9)	0.046	0.047	0.3 (0.1–0.9)	0.047	0.054	0.6 (0.1–2.1)	0.513	0.526
rs224446_t	*SLC11A2*	0.7 (0.4–1.0)	0.047	0.047	0.7 (0.4–1.2)	0.222	0.233	0.9 (0.4–2.0)	0.899	0.897
rs16966334_g	*B2M*	2.4 (1.3–4.2)	0.003	0.006	1.3 (0.3–4.7)	0.708	0.726	2.8 (1.3–6.0)	0.008	0.004
rs1901531_c	*B2M*	1.6 (1.1–2.5)	0.028	0.023	2.2 (1.3–3.8)	0.004	0.006	0.5 (0.1–1.3)	0.262	0.265

The *p-*values (*p*) shown were obtained by multivariable logistic regression, adjusting for age, total D-drug exposure, CD4^+^ T-cell nadir, plasma HIV RNA concentration, all 4 principal component ancestry variables, and self-reported race (if not race-stratified) and by permutation analysis (empiric *p*-value, *p*_emp_). *Abbreviations: OR*, odds ratio; *95% CI*, 95% Confidence Interval; *TF*, transferrin; *CP*, ceruloplasmin; *TFRC*, transferrin receptor 1; *BMP6*, bone morphogenetic protein 6; *ACO1*, cytoplasmic aconitase; *SLC11A2*, divalent metal transporter 1; *B2M*, beta-2 microglobulin.

The strong association of SNP rs2026739 in *ACO1* with DNP in whites by both logistic regression and permutation analysis remained statistically significant after adjustment for multiple testing (*p_emp_<*0.0001). Notably, no linkage disequilibrium was observed between statistically significant SNPs and other SNPs within the same genes, as exemplified in the LD maps for *CP* and *ACO1* (SNPs rs3816893 and rs2026739), shown in Figure 1.

The prevalence of ferromic variants was compared across three categories of severity of DNP, which was graded as *none* (0); *slight or mild* (1–2); and *moderate to severe* (3–4) in race-stratified analyses (Table 4). Only associations with *p*-values≤0.05 are presented. Polymorphisms in *ACO1* (rs2026739), *B2M* (rs16966334 and rs1901531), *CP* (rs3816893), *TF* (rs2718796 and rs8177306), and *TFRC* rs480760, most of which are very common among both blacks and whites, showed statistically significant associations with DNP severity in one or both subgroups as well as in the entire study population.

**Table 4:** Iron-transport-related genes associated with severity of distal neuropathic pain in CHARTER.

Gene/SNP	Neuropathic Pain Severity (n,%)^1^			All subjects N = 558	Blacks N = 242	Whites N = 247	Allele Frequency^3^ Overall (B/W)
	None	Slight/Mild	Mod/Severe	*p*-value for trend^2^			
***ACO1***							
rs2026739_g	209 (54)	76 (66)	38 (73)	0.013	0.148	0.002	0.46 (0.54/0.36)
rs7033149_g	133 (34)	52 (44)	24 (46)	0.044	0.089	0.222	0.20 (0.30/0.14)
rs4495514_t	39 (10)	3 (3)	4 (8)	0.027	0.111	---4	0.05 (0.11/---)
***B2M***							
rs16966334_g	37 (10)	20 (17)	7 (13)	0.027	0.009	0.618	0.06 (0.08/0.02)
rs1901531_c	71 (18)	29 (25)	16 (31)	0.009	0.316	0.005	0.12 (0.04/0.21)
**** ***BMP6***							
rs267202_a	258 (67)	68 (59)	24 (47)	0.008	0.078	0.206	0.57 (0.57/0.50)
rs376308_g	243 (63)	59 (51)	23 (44)	0.014	0.203	0.315	0.49 (0.58/0.35)
***CP***							
rs9853335_c	38 (10)	16 (15)	10 (20)	0.034	0.758	0.124	0.06 (0.02/0.11)
rs3816893_t	65 (17)	32 (27)	10 (19)	0.024	0.011	0.210	0.10 (0.12/0.10)
***FXN***							
rs3793451_t	46 (12)	7 (6)	4 (8)	0.051	0.289	0.094	0.06 (0.04/0.04)
***TF***							
rs2718796_g	14 (3)	5 (4)	9 (17)	0.004	0.154	0.009	0.03 (0.02/0.02)
rs8177306_g	54 (14)	7 (6)	4 (8)	0.009	0.026	---4	0.08 (0.18/---)
***TFRC***							
rs480760_t	110 (28)	22 (19)	8 (15)	0.005	0.038	0.476	0.19 (0.37/0.05)

1 Numbers of subjects shown are for entire study population, including 10.4% non-Hispanic Black and 2.1% “Other” self-reported race/ethnicity.

2 *P*-values presented were obtained using a non-parametric test for trend.

3 Allele frequency in entire CHARTER study population.

4 Allele not present in whites.

*Abbreviations:* Mod/Sev, moderate to severe neuropathic pain; B/W, Non-Hispanic Blacks/Whites; *ACO1*, cytoplasmic aconitase (iron-regulatory protein 1); *B2M*, beta-2 microglobulin; *BMP6*, bone morphogenetic protein-6; *CP*, ceruloplasmin; *TF*, transferrin; *TFRC*, transferrin receptor 1.

## Discussion

This represents the first study to associate key iron-regulatory and iron-transport-pathway genes, which make up the human ferrome and are critical for maintenance of neuronal metabolism and mitochondrial function, with painful neuropathy in HIV-infected subjects. Our results particularly support the concept that iron metabolism plays a role in the pathophysiology of DNP for the following reasons: 1) the same genomic variants showed associations with DNP by conventional regression and by permutation-based analytical methods, which account for the effects of multiple statistical tests, gene size, and linkage disequilibrium; 2) thirteen of the same SNPs (in 7 genes) were also associated with the *severity* of DNP in this population; and 3) point estimates for ORs were consistent in direction and very similar in magnitude in both blacks and whites, as might be anticipated for valid, fundamental biological associations. Furthermore, ACO1 SNP rs2026739 retained its statistically significant association with DNP after adjustment for multiple testing. Our observation that associations of certain SNPs (*e.g*., in *CP*, *ACO1*, *TF*, and *B2M* genes) with DNP and with DNP severity predominate in either blacks or whites may in part explain reported population differences with regard to pain threshold and pain-related disability, albeit in non-HIV-infected populations [41], [42]. Interaction with race-specific genomic and lifestyle factors within these population subgroups is also possible. With the exception of *BMP6*, none of the genes we associated with DNP occurs on chromosome 6*p*, indicating that genomic variation in iron metabolism and transport *per se*, not linkage disequilibrium with HLA Class I-linked haplotypes that modulate inflammation, is likely to be responsible for the observed associations.

Several lines of evidence support a role for iron metabolism in the development of HIV-SN and/or DNP, which remain significant problems even in the era of newer-generation cART [43], [44]. Common variants in the iron-loading *HFE* gene were associated with reduced risk of HIV-SN during mitochondrial-toxic cART regimens in AIDS Clinical Trial Group Study 384, and the protective association with *HFE* was replicated in this study [31]. HIV infection itself induces cellular iron dysregulation, possibly via downregulation of macrophage Hfe expression by the viral Nef protein and induction of the pro-inflammatory, iron-regulatory hormone hepcidin [45]–[47]. Restless legs syndrome (RLS), a disorder characterized by unpleasant sensations in the lower extremities that are relieved by movement, has been reported in some studies to be more prevalent in HIV-infected individuals with painful neuropathy and has been likened to a unique form of neuropathic pain [48], [49]. Systemic iron deficiency, brain iron deficiency, and activation of the hypoxia-inducible factor pathway have been linked to the development of RLS, and in a significant subset of patients, iron supplementation alleviates symptoms [50]–[52]. The biological plausibility of a role for iron metabolism in the pathophysiology of inflammatory peripheral neuropathy and DNP is also suggested by prior experimental studies and growing appreciation within the field of iron metabolism of the importance of iron transport and its tight regulation to maintenance of energy metabolism in highly metabolically active cells such as neurons, and to immune regulation [27], [47], [53]–[57]. Iron transport is critical to neuronal energy metabolism and axoplasmic transport [58]–[60]. Inhibitors of heme oxygenase, which catalyzes the breakdown of heme to iron, biliverdin and carbon monoxide, have been shown to have analgesic effects in some models of inflammatory or neuropathic pain [61]. Genetic variation in iron transport has also been associated with altered inflammatory cytokine levels, which at least in part mediate neuropathic pain [62]–[66]. Involvement of bone morphogenetic proteins in pain perception and sensitivity has also been reported in rodent models [67].

The *ACO1* and *FXN* genes are important for cellular energy metabolism and mitochondrial iron transport: *ACO1* encodes a bifunctional protein containing an iron-sulfur cluster that reversibly binds to regulatory elements in transferrin receptor (Tfr) and ferritin mRNAs depending on ambient cellular iron levels [55]. This protein functions as a cytoplasmic aconitase under low-iron conditions, upregulating synthesis of Tfr (the major cellular iron importer) by stabilizing Tfr mRNA, while preventing translation of ferritin (the principal iron storage protein in the cell); the reverse occurs when ambient iron levels are high [68]. The *FXN* gene, which is mutated in Friedreich's ataxia, regulates mitochondrial iron utilization and export; a defect in the encoded frataxin molecule in this disease results in progressive mitochondrial iron accumulation and oxidative injury in a variety of tissues with high metabolic demand, including the heart and central nervous system [69]–[71]. Beta-2 microglobulin is a ubiquitous iron- and Hfe-stabilizing protein which also promotes nerve repair following injury, and it is a marker of disease progression in a variety of diseases, but it has not previously been associated with DNP [72]. The *CP* rs3816893 and rs1302552 SNPs, which were associated with DNP alone in this population, also showed strong associations with the presence of neuropathy symptoms in general, which included paresthesias and loss of sensation. These SNP associations also met multiple-testing criteria for significance after permutation analysis, lending support to a role for *CP* in DNP. Ceruloplasmin is a ferroxidase of critical importance to neuronal copper and iron regulation and in neuronal protection against iron-mediated oxidative injury in the central and peripheral nervous systems [73]. The non-synonymous *CP* SNP rs13072552 has been associated in genome-wide association studies with higher serum ceruloplasmin levels [74]. While serum ceruloplasmin is an acute-phase reactant (levels rise during acute inflammation), serum ceruloplasmin levels may also be a marker of oxidative neuronal injury, as in diabetic neuropathy.[75]. Disturbances in ceruloplasmin homeostasis have been linked to other neurodegenerative diseases, and to neuropathic pain in the constriction injury rat model of neuropathic pain.[76]. Similarly, transferrin levels have been associated with peripheral neuropathy after bariatric surgery, which has been shown to disrupt iron homeostasis [77].

Iron transport in all tissues is intricately balanced with cellular and mitochondrial iron demand; indeed, recent studies including our own suggest that iron levels regulate mitochondrial biogenesis, a determinant of mitochondrial function [78]. (*Kallianpur et al, unpublished data*) Therefore, it should not be surprising if altered iron transport increases susceptibility to HIV-SN and DNP, especially in individuals exposed to mitochondria-toxic forms of cART. Mitochondrial injury, as well as activation of glia in the spinal cord and dorsal root ganglia due to HIV envelope glycoprotein gp120, with consequent release of inflammatory cytokines, may be involved in the pathogenesis of DNP [79]. Finally, others have reported that dysregulated iron transport also alters glutamate metabolism, which is implicated in both drug-induced and non-toxic peripheral neuropathies [80]–[82].

This study has some acknowledged limitations. The functional effects of these SNPs in iron-related genes are currently unknown. Since regulation of iron metabolism occurs largely at the post-transcriptional level, we speculate that many of these variants alter gene regulation and protein levels rather than protein structure or function. Assay of serum protein levels was not possible as part of this study, but *CP* rs13072552 has been linked to increased ceruloplasmin levels [74], and several of the genes identified here encode measurable serum proteins that may be investigated in future studies. The functional relatedness of the genes we selected in iron metabolism, in addition to tightly coordinated regulation of some of these genes (*e.g.*, *ACO1* and *TFRC*) in order to fine tune cellular iron access, storage, and utilization, argue against reliance on genome-wide levels of significance or stringent multiple-testing corrections in an exploratory study of this type; individual iron-regulatory SNP genotypes are highly unlikely to be independent, as represented in Figure 1. In this situation, correction for the number of genes analyzed may be a more logical approach: with this type of correction, *TF* rs2718796, *CP* rs3816893, and *B2M* rs16966334, as well as *ACO1* rs2026739 in whites, remain statistically significant or nominally significant. (As noted, the *ACO1* SNP also remained significant after Bonferroni correction for multiple tests.) We did not adjust in our analyses for height or alcohol consumption, factors which can influence susceptibility to neuropathy, but not necessarily DNP. However, alcohol consumption was not associated with DNP on univariable analysis. Information about hair color, which has been correlated with neuropathic pain sensitivity, was not available [83]. We also attempted to address the potential effect of diabetes on genetic associations with peripheral neuropathy and DNP in this population. Glycosylated hemoglobin values were not available for analysis on most subjects, but additional adjustment for the possible presence of treated or untreated diabetes, ascertained using a more stringent-than-normal cut-off for non-fasting blood glucose values, did not significantly impact our results. Finally, the purpose of this study was to build on our prior findings of iron-related genetic associations with HIV-SN and to test the hypothesis that iron metabolism, rather than inflammation alone, plays a role in HIV-associated DNP; we intentionally did not study an exhaustive list of iron-regulatory genes. Much of the ferrome therefore remains to be studied. Keltner et al recently reported that HIV-associated DNP is associated in CHARTER subjects with reduced total cortical gray matter volumes on neuroimaging; cortical gray matter volumes have also been linked to R2* relaxation times, a sensitive measure of brain iron content [84], [85].

In summary, SNPs in eight iron-related genes selected for analysis showed significant and biologically plausible associations with DNP, independent of known potential confounders; SNPs in 7 of these genes also predicted DNP severity, adding to the biological plausibility argument. Most importantly, the common ACO1 rs2026739 variant remained significantly associated with DNP and DNP severity after stringent correction for multiple tests. Furthermore, associations with DNP were relatively robust, and point estimates for SNP associations were similar in direction and magnitude in blacks and whites, if not statistically significant in both groups. These results support the concept that the ferrome may be involved in the pathogenesis of HIV-SN and DNP in HIV-infected individuals on cART. These new genetic associations will require replication in other population samples as well as further functional characterization to determine underlying biological mechanisms that may be therapeutically targeted.

## Supporting Information

Figure S1 **Minimal overlap between ancestry principal components (PCs).** PCs plotted against one another demonstrate appropriate clustering, with few outliers. *Black dots*: black; *blue dots:* Hispanic; *red dots:* white; *cyan dots:* other.
(TIF)

Table S1 **Iron-related genes and variants evaluated in relation to neuropathy in CHARTER.**
(PDF)
