# Supplementary figures and images for: Genetic Variation in Iron Metabolism Is Associated with Neuropathic Pain and Pain Severity in HIV-Infected Patients on Antiretroviral Therapy
Source: PLoS One. 2014 Aug 21;9(8):e103123. doi: 10.1371/journal.pone.0103123 (PMC4140681; doi:10.1371/journal.pone.0103123)

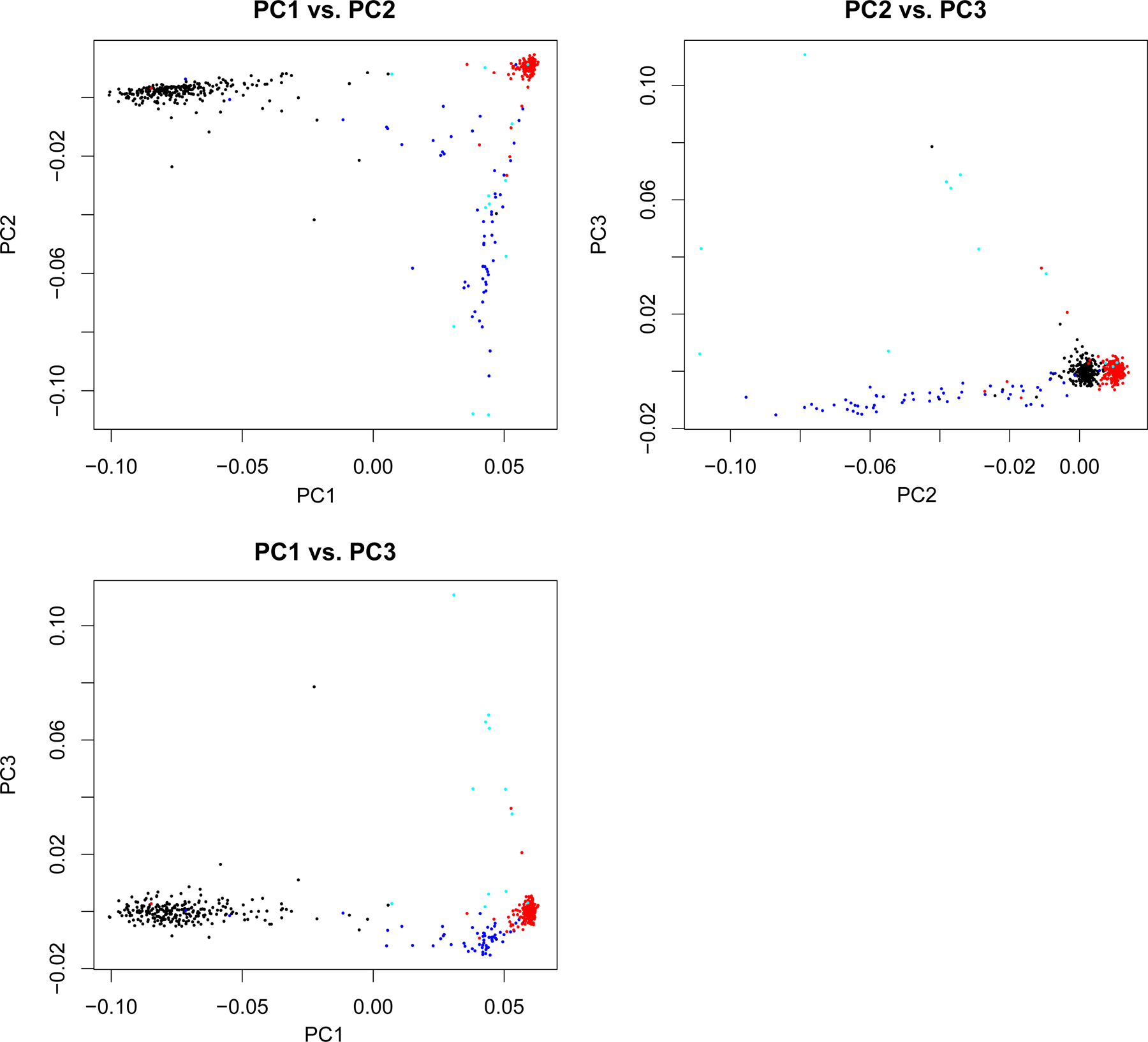

Supplement: Figure S1 — Minimal overlap between ancestry principal components (PCs). PCs plotted against one another demonstrate appropriate clustering, with few outliers. Black dots: black; blue dots: Hispanic; red dots: white; cyan dots: other. (TIF) [file pone.0103123.s001.tif]
