# Supplementary material for: Genetic Variation in Iron Metabolism Is Associated with Neuropathic Pain and Pain Severity in HIV-Infected Patients on Antiretroviral Therapy
Source: PLoS One. 2014 Aug 21;9(8):e103123. doi: 10.1371/journal.pone.0103123 (PMC4140681; doi:10.1371/journal.pone.0103123)
Supplement: Table S1 — Iron-related genes and variants evaluated in relation to neuropathy in CHARTER. (PDF) [file pone.0103123.s002.pdf]

Gene	SNP rs number	Chromosome	Probe Set ID on Affymetrix Genome-Wide SNP array 6.0
HFE	rs2071303	6	SNP_A-4273092
HFE	rs707889	6	SNP_A-2150639
HFE	rs2858996	6	SNP_A-8440531
HFE	rs1800562	6	SNP_A-1905121
SLC40A1	rs13404407	2	SNP_A-8481584
SLC40A1	rs1123109	2	SNP_A-8475616
SLC40A1	rs11568351	2	SNP_A-4293724
SLC40A1	rs4667287	2	SNP_A-2027694
SLC40A1	rs3792079	2	SNP_A-8321976
SLC40A1	rs2352262	2	SNP_A-8602895
SLC40A1	rs930373	2	SNP_A-4217598
SLC40A1	rs1123110	2	SNP_A-4218292
SLC40A1	rs11568350	2	SNP_A-8354595
SLC40A1	rs2304704	2	SNP_A-2027959
SLC11A1	rs2276631	2	SNP_A-8369368
SLC11A1	rs2695343	2	SNP_A-2095550
SLC11A1	rs13062	2	SNP_A-1950973
SLC11A1	rs7576974	2	SNP_A-1788623
HFE2	rs7540883	1	SNP_A-8652915
HFE2	rs10218795	1	SNP_A-2259589
HAMP	rs10414846	19	SNP_A-2139930
HAMP	rs10421768	19	SNP_A-4276731
HAMP	rs8101606	19	SNP_A-8449520
TF	rs8177232	3	SNP_A-2209195
TF	rs8177224	3	SNP_A-8440993
TF	rs1799852	3	SNP_A-1931871
TF	rs8177248	3	SNP_A-8565154
TF	rs2715627	3	SNP_A-1913594
TF	rs8177262	3	SNP_A-8647674
TF	rs8177203	3	SNP_A-1841130
TF	rs8177220	3	SNP_A-4195301
TF	rs8177191	3	SNP_A-8657424
TF	rs8177277	3	SNP_A-2283755
TF	rs3811657	3	SNP_A-8708020
TF	rs1525892	3	SNP_A-8618559
TF	rs8177238	3	SNP_A-4237109
TF	Rs8177272	3	SNP_A-A4280184

Gene	SNP rs number	Chromosome	Probe Set ID on Affymetrix Genome-Wide SNP array 6.0
TF	rs2715632	3	SNP_A-4277378
TF	rs2718796	3	SNP_A-8335105
TF	rs2715631	3	SNP_A-2241618
TF	rs6778321	3	SNP_A-2147949
TF	rs8177253	3	SNP_A-8406526
TF	rs8177306	3	SNP_A-1883149
TF	rs7645538	3	SNP_A-8654418
TF	rs8177235	3	SNP_A-2306486
TF	rs8177215	3	SNP_A-8675996
TF	rs1800277	3	SNP_A-2182363
TF	rs1049296	3	SNP_A-1891036
TFRC	rs3933	3	SNP_A-1956928
TFRC	rs4927866	3	SNP_A-8371201
TFRC	rs17788379	3	SNP_A-8572471
TFRC	rs17091382	3	SNP_A-8549536
TFRC	rs12330245	3	SNP_A-8625844
TFRC	rs17788373	3	SNP_A-8412514
TFRC	rs3804141	3	SNP_A-1975790
TFRC	rs17091378	3	SNP_A-1855480
TFRC	rs480760	3	SNP_A-8620139
TFR2	rs4521695	7	SNP_A-8447015
BMP2	rs7270163	20	SNP_A-2280450
BMP2	rs1979855	20	SNP_A-2114967
BMP2	rs235767	20	SNP_A-8561610
BMP2	rs6107869	20	SNP_A-2082410
BMP6	rs10498672	6	SNP_A-8666903
BMP6	rs911753	6	SNP_A-4200956
BMP6	rs1225924	6	SNP_A-8297217
BMP6	rs4371882	6	SNP_A-4203185
BMP6	rs169123	6	SNP_A-8551124
BMP6	rs267190	6	SNP_A-8666907
BMP6	rs267170	6	SNP_A-8428785
BMP6	rs267202	6	SNP_A-1984984
BMP6	rs267806	6	SNP_A-2088998
BMP6	rs267186	6	SNP_A-2237158
BMP6	rs13196371	6	SNP_A-8388290
BMP6	rs6938135	6	SNP_A-1831240
BMP6	rs270398	6	SNP_A-2098774

Gene	SNP rs number	Chromosome	Probe Set ID on Affymetrix Genome-Wide SNP array 6.0
BMP6	rs270374	6	SNP_A-2130301
BMP6	rs270377	6	SNP_A-1849374
BMP6	rs270400	6	SNP_A-2113463
BMP6	rs881891	6	SNP_A-8468880
BMP6	rs1235192	6	SNP_A-8478865
BMP6	rs270397	6	SNP_A-8608073
BMP6	rs267807	6	SNP_A-2067023
BMP6	rs911749	6	SNP_A-1828739
BMP6	rs7768784	6	SNP_A-8666908
BMP6	rs270383	6	SNP_A-2313678
BMP6	rs911750	6	SNP_A-4294782
BMP6	rs267840	6	SNP_A-1857627
BMP6	rs11960967	6	SNP_A-8522587
BMP6	rs9379137	6	SNP_A-1829613
BMP6	rs267196	6	SNP_A-2169074
BMP6	rs270388	6	SNP_A-8465811
BMP6	rs267207	6	SNP_A-1922269
BMP6	rs376308	6	SNP_A-8401116
BMP6	rs12215656	6	SNP_A-8446085
BMP6	rs1226102	6	SNP_A-8426870
BMP6	rs1358892	6	SNP_A-1950172
BMP6	rs267203	6	SNP_A-2257015
BMP6	rs1226101	6	SNP_A-2011551
BMP6	rs267205	6	SNP_A-2108180
BMP6	rs267184	6	SNP_A-1909474
BMP6	rs6919067	6	SNP_A-2221499
BMP6	rs1043784	6	SNP_A-8377968
BMP6	rs1225929	6	SNP_A-8666909
BMP6	rs267206	6	SNP_A-4291007
BMP6	rs267183	6	SNP_A-8666906
BMP6	rs13212191	6	SNP_A-8615178
BMP6	rs13192135	6	SNP_A-1917742
BMP6	rs267195	6	SNP_A-4194688
BMP6	rs6913143	6	SNP_A-8418101
BMP6	rs1107495	6	SNP_A-1891031
BMP6	rs11243204	6	SNP_A-2236932
BMP6	rs2876117	6	SNP_A-2035994
BMP6	rs2116238	6	SNP_A-8641411
BMP6	rs7768988	6	SNP_A-8433147
BMP6	rs267180	6	SNP_A-8507535
BMP6	rs17673852	6	SNP_A-8518107
Slc11A2	rs17125212	12	SNP_A-1880618
Slc11A2	rs224446	12	SNP_A-2067039
Slc11A2	rs2269683	12	SNP_A-1945877

Gene	SNP rs number	Chromosome	Probe Set ID on Affymetrix Genome-Wide SNP array 6.0
Slc11A2	rs224573	12	SNP_A-4218203
Slc11A2	rs150909	12	SNP_A-2058555
Slc11A2	rs224568	12	SNP_A-8457497
Slc11A2	rs224589	12	SNP_A-8510392
Slc11A2	rs224572	12	SNP_A-2058598
CP	rs773050	3	SNP_A-8654751
CP	rs772908	3	SNP_A-2023463
CP	rs17787768	3	SNP_A-1786245
CP	rs1879169	3	SNP_A-8570079
CP	rs16861577	3	SNP_A-8393392
CP	rs4974389	3	SNP_A-2179747
CP	rs17838831	3	SNP_A-2235980
CP	rs16861598	3	SNP_A-2064503
CP	rs16861634	3	SNP_A-2167565
CP	rs16861590	3	SNP_A-2178014
CP	rs13072552	3	SNP_A-8319180
CP	rs9853335	3	SNP_A-8563286
CP	rs11924961	3	SNP_A-8715775
CP	rs13075921	3	SNP_A-8465773
CP	rs16861579	3	SNP_A-8680655
CP	rs3816893	3	SNP_A-2009215
CP	rs701755	3	SNP_A-2304646
FXN	rs2498430	9	SNP_A-8470004
FXN	rs7860403	9	SNP_A-2276496
FXN	rs3793451	9	SNP_A-8363027
FXN	rs4744787	9	SNP_A-8644254
FXN	rs7047274	9	SNP_A-4292586
FXN	rs2498419	9	SNP_A-8522000
FXN	rs2498431	9	SNP_A-8292147
FXN	rs17060788	9	SNP_A-2064166
FXN	rs12001326	9	SNP_A-1946335
FXN	rs11145043	9	SNP_A-8630238
FXN	rs9411170	9	SNP_A-8345218
FXN	rs1411675	9	SNP_A-8283077
FXN	rs2498434	9	SNP_A-2134314
FXN	rs7870295	9	SNP_A-4255749
FXN	rs7871596	9	SNP_A-8453359
FXN	rs2498432	9	SNP_A-8621987
FXN	rs2309393	9	SNP_A-8401321
FTMT	rs13358715	5	SNP_A-8361993
FTH1	rs17156609	11	SNP_A-8365828

FTH1	rs195154	11	SNP_A-8600815

Gene	SNP rs number	Chromosome	Probe Set ID on Affymetrix Genome-Wide SNP array 6.0
ATP13A2	rs3738815	1	SNP_A-2090089
ATP13A2	rs6684770	1	SNP_A-2146554
ACO1	rs13302577	9	SNP_A-8345067
ACO1	rs10813818	9	SNP_A-1951915
ACO1	rs10970972	9	SNP_A-8553411
ACO1	rs7033149	9	SNP_A-8547242
ACO1	rs16918276	9	SNP_A-2125665
ACO1	rs7022554	9	SNP_A-8391453
ACO1	rs10738885	9	SNP_A-2093675
ACO1	rs10813816	9	SNP_A-1925443
ACO1	rs10435797	9	SNP_A-1869485
ACO1	rs13292540	9	SNP_A-8709558
ACO1	rs7866419	9	SNP_A-8373829
ACO1	rs3780473	9	SNP_A-4267458
ACO1	rs4879583	9	SNP_A-8457046
ACO1	rs1028932	9	SNP_A-1868464
ACO1	rs2026739	9	SNP_A-1955609
ACO1	rs4495514	9	SNP_A-8324358
ACO1	rs10970974	9	SNP_A-8653123
ACO1	rs13293491	9	SNP_A-1930244
ACO1	rs3780474	9	SNP_A-4267457
ACO1	rs7019520	9	SNP_A-8688496
ACO1	rs7032871	9	SNP_A-2040065
ACO2	rs6002390	22	SNP_A-8412401
ACO2	rs5751114	22	SNP_A-8391252
ACO2	rs9611598	22	SNP_A-8653122
B2M	rs2254835	15	SNP_A-4242772
B2M	rs1690313	15	SNP_A-8602380
B2M	rs1901531	15	SNP_A-8418019
B2M	rs16966334	15	SNP_A-8527362
B2M	rs16966424	15	SNP_A-1894070

Table 1. Iron-related genes and variants evaluated in relation to neuropathy in CHARTER.
